# Irrigation and phosphorus fertilization regulate trade-offs among yield, forage quality, and resource-use efficiency in alfalfa production under arid and semi-arid conditions

**DOI:** 10.3389/fpls.2026.1885390

**Published:** 2026-07-29

**Authors:** Yindi Wang, Rui Zhang, Peixun Liu, Guangyuan Ma, Hongjuan Zhang

**Affiliations:** 1 College of Water Conservancy and Hydropower Engineering, Gansu Agricultural University, Lanzhou, China; 2 Gansu Provincial Agricultural Smart Water-Saving Technology Innovation Center, Lanzhou, Gansu, China

**Keywords:** alfalfa, forage quality, irrigation water use efficiency, phosphorus agronomic efficiency, soil available nutrients, water–phosphorus coupling

## Abstract

**Introduction:**

Optimizing water–phosphorus coupling is essential for resolving the trade-offs among high yield, improved forage quality, efficient resource use, and soil nutrient sustainability in alfalfa production in arid and semi-arid regions. Yet, how irrigation regimes and phosphorus fertilization jointly regulate these interconnected agronomic, physiological, quality-related, and soil nutrient responses remains unclear.

**Methods:**

A two-year field experiment was during the 2024 and 2025 growing seasons, using three irrigation regimes: W1, moderate water stress; W2, mild water stress; and W3, full irrigation, combined with three phosphorus levels: P0, P50, and P100. Alfalfa yield, irrigation water use efficiency (IWUE), phosphorus agronomic efficiency (AEP), plant morphology, forage quality, leaf photosynthetic traits, and soil available nutrients in the 0–60 cm soil profile were measured across three cuttings. Principal component analysis and correlation analysis were used to evaluate the integrated effects of water–phosphorus coupling.

**Results:**

Irrigation, phosphorus fertilization, and year significantly affected alfalfa yield, IWUE, plant morphology, and photosynthetic characteristics, while their interactions significantly influenced yield, IWUE, agronomic phosphorus efficiency (AEP), plant morphology, forage quality, and photosynthetic characteristics, as well as soil nutrient status, whereas interaction effects varied among indicators. Across treatments, W3P50 produced the highest yield, with no significant difference from W3P100. Under P50, W2P50 and W3P50 increased yield by 5.3% and 7.9%, respectively, compared with W1P50, while under P100, W2P100 and W3P100 increased yield by 4.0% and 5.0%, respectively, compared with W1P100. Under W2 and W3, P50 and P100 produced significantly higher IWUE than P0, but no significant difference was observed between P50 and P100, indicating that moderate phosphorus input was sufficient under improved irrigation conditions. W2P50 and W3P50 showed higher AEP than W1P50 and W3P100. W2P50 and W3P50 also significantly increased crude protein content compared with W1P50. Full irrigation enhanced Pn, Tr, and Gs, while W2P50 and W3P50 maintained higher intrinsic water-use efficiency (WUEi). The entropy-weighted TOPSIS evaluation showed that W2P50 had the highest comprehensive score, followed by W3P50 and W2P100.

**Discussion:**

Water–phosphorus coupling regulated alfalfa production by coordinating soil nutrient availability, photosynthetic capacity, plant growth, forage quality, and resource-use efficiency. W2P50 showed the best overall performance by maintaining stable yield while improving AEP, IWUE, and forage quality, and is therefore recommended as an optimized water-saving and phosphorus-efficient strategy.

## 1 Introduction

Alfalfa (*Medicago sativa* L.) is one of the most widely cultivated perennial legume forages worldwide. It is grown in more than 80 countries, with a global planting area of approximately 35 million ha, and is regarded as one of the most important legume species in forage production systems (Kayad et al., 2016). Alfalfa is characterized by high crude protein concentration, good palatability, strong regrowth capacity, high forage yield, and broad ecological adaptability, making it essential for livestock production and high-quality forage supply (Kamran et al., 2022). As a representative legume forage, alfalfa can increase soil nitrogen inputs through symbiotic biological nitrogen fixation, thereby reducing dependence on external nitrogen fertilizers. In addition, its rapid growth and strong post-cutting regrowth capacity allow multiple harvests within a growing season, making it a key forage species for developing water-saving forage–livestock systems in arid and semi-arid regions (Wang et al., 2023). Although alfalfa shows relatively strong environmental adaptability, its yield formation, forage quality, and plant growth are highly sensitive to water and nutrient supply (Liu et al., 2021a). In regions such as the Yinda Irrigation District of Gansu Province, where water resources are limited and soils often exhibit salinization and alkalization characteristics, insufficient water supply and low phosphorus availability have become major constraints to high-yielding and high-quality alfalfa production (Sun et al., 2025).

Water availability is a key factor regulating alfalfa growth, development, and yield formation (Djaman et al., 2020). Adequate soil moisture helps maintain leaf water status and stomatal opening, thereby facilitating CO_2_ diffusion into mesophyll cells and enhancing net photosynthetic rate, transpiration rate, and photosynthate accumulation. These physiological processes provide the basis for plant morphological development, including plant height, stem diameter, branch number, and leaf-to-stem ratio (Abid et al., 2016). In contrast, water stress induces stomatal closure, reduces stomatal conductance and intercellular CO_2_ supply, and subsequently suppresses photosynthesis and dry matter accumulation, ultimately leading to restricted plant growth, yield reduction, and changes in forage quality (Liu et al., 2018). Previous studies in the inland arid regions of central and western China have shown that alfalfa dry matter yield decreases markedly with reduced irrigation. For example, under full-season irrigation, dry matter yield in 2018 decreased from 31.1 to 20.3 t ha^-1^ under flood irrigation and from 22.1 to 12.3 t ha^-1^ under subsurface drip irrigation, indicating that inadequate water supply strongly limits alfalfa yield formation (Liu et al., 2021a). Meanwhile, appropriate deficit irrigation can maintain relatively high productivity under water-saving conditions. Liu et al. (2021) reported that alfalfa yield reached up to 34.9 t ha^-1^, and irrigation water use efficiency under subsurface drip irrigation ranged from 13.8 to 84.4 kg ha^-1^ mm^-1^, which was higher than that under flood irrigation, ranging from 10.8 to 66.6 kg ha^-1^ mm^-1^. In addition, deficit irrigation has been proposed as an important strategy for saving water in water-limited alfalfa systems. For instance, in water-scarce areas of California, mid-summer deficit irrigation reduced crop water use by 224–239 mm, although it also caused a certain degree of yield reduction, indicating a clear trade-off between water saving and forage production (Hanson et al., 2007). These findings suggest that irrigation method and irrigation amount strongly affect alfalfa hay yield, forage nutritive value, water use efficiency, and water-saving benefits (Li et al., 2023). However, excessive irrigation does not necessarily sustain further growth improvement. Instead, it may reduce soil aeration, restrict deep root development, and increase risks of water waste and nutrient leaching (Liu et al., 2021b). Therefore, identifying an appropriate irrigation level under drip irrigation in the Yinda Irrigation District is essential for achieving high yield, high forage quality, and high irrigation water use efficiency in alfalfa production (Zheng et al., 2023).

Phosphorus is an essential macronutrient for alfalfa growth and development, and it participates in key physiological processes such as energy metabolism, nucleic acid synthesis, root development, photosynthate transport, and symbiotic nitrogen fixation (Marschner, 2012). Appropriate phosphorus fertilization can promote root growth, improve root capacity for water and nutrient uptake, enhance leaf photosynthetic performance, and increase dry matter accumulation, thereby improving hay yield (Berg et al., 2005). Phosphorus supply also affects forage quality formation. Adequate phosphorus application can increase crude protein concentration, improve neutral detergent fiber and acid detergent fiber characteristics, and enhance the feeding value of alfalfa (Zhang et al., 2025). Previous studies have shown that under drip irrigation, phosphorus application at 100 kg ha^-1^ can improve water and phosphorus use efficiencies and enhance alfalfa productivity and nutritive quality (Zhang et al., 2020a, 2020b). Other studies have reported that phosphorus fertilization can significantly increase plant height and branch number in salt-affected soils, with better growth performance observed at 180 kg ha^-1^ phosphorus application (Tian et al., 2025). These results indicate that alfalfa has a strong demand for phosphorus, but the growth-promoting effect of phosphorus occurs within an appropriate range. Insufficient phosphorus supply can limit root development, photosynthesis, and biological nitrogen fixation, whereas excessive phosphorus application may not further increase plant growth and may instead reduce phosphorus use efficiency, promote soil phosphorus accumulation, and increase environmental risks (Gu et al., 2018). For example, Hao et al. (2025) found that shoot growth of alfalfa no longer increased markedly when phosphorus supply exceeded 40 mg P kg^-1^ soil in acidic soils, suggesting that the positive effect of phosphorus on alfalfa growth has a threshold response.

The effects of water and phosphorus on alfalfa growth are not independent but are strongly interactive (Kong et al., 2020). On the one hand, soil moisture influences phosphorus diffusion, transport, and root uptake in soil, and suitable water conditions can improve soil phosphorus availability (Gu et al., 2018). On the other hand, phosphorus can promote root development and root activity, thereby enhancing water uptake and utilization and alleviating the negative effects of water stress on plant growth (Fan et al., 2016). Therefore, appropriate irrigation combined with suitable phosphorus fertilization may synergistically improve alfalfa yield, irrigation water use efficiency, and forage quality by increasing soil available nutrient supply, enhancing leaf photosynthetic capacity, optimizing plant morphological development, and promoting dry matter accumulation (Liu et al., 2021b). However, in arid and semi-arid irrigation districts, increasing irrigation may enhance alfalfa yield and photosynthetic capacity, but it may also reduce irrigation water use efficiency. Similarly, higher phosphorus inputs may not continuously increase yield when water supply is adequate. Thus, water–phosphorus management may involve a trade-off among yield, forage quality, and resource-use efficiency.

Based on this background, this study aimed to clarify how irrigation regimes and phosphorus fertilization interactively regulate alfalfa productivity, forage quality, resource-use efficiency, and soil available nutrient status under arid and semi-arid conditions. We hypothesized that: (1) increasing irrigation to an appropriate level would promote soil available nutrient supply and leaf photosynthetic capacity, thereby enhancing plant morphological development and yield formation, whereas full irrigation would reduce irrigation water use efficiency; (2) phosphorus fertilization would improve alfalfa growth, photosynthetic characteristics, and forage quality, but under mild water stress or full irrigation, moderate phosphorus application would be sufficient to meet alfalfa production requirements, and further phosphorus addition would provide limited benefits for yield and water use efficiency; and (3) mild water stress combined with moderate phosphorus application would maintain relatively high yield while improving irrigation water use efficiency, phosphorus agronomic efficiency, and forage quality, representing an optimized water–phosphorus combination for stable yield, high quality, and efficient resource use.

## 2 Materials and methods

### 2.1 Study site

**Figure 1:**
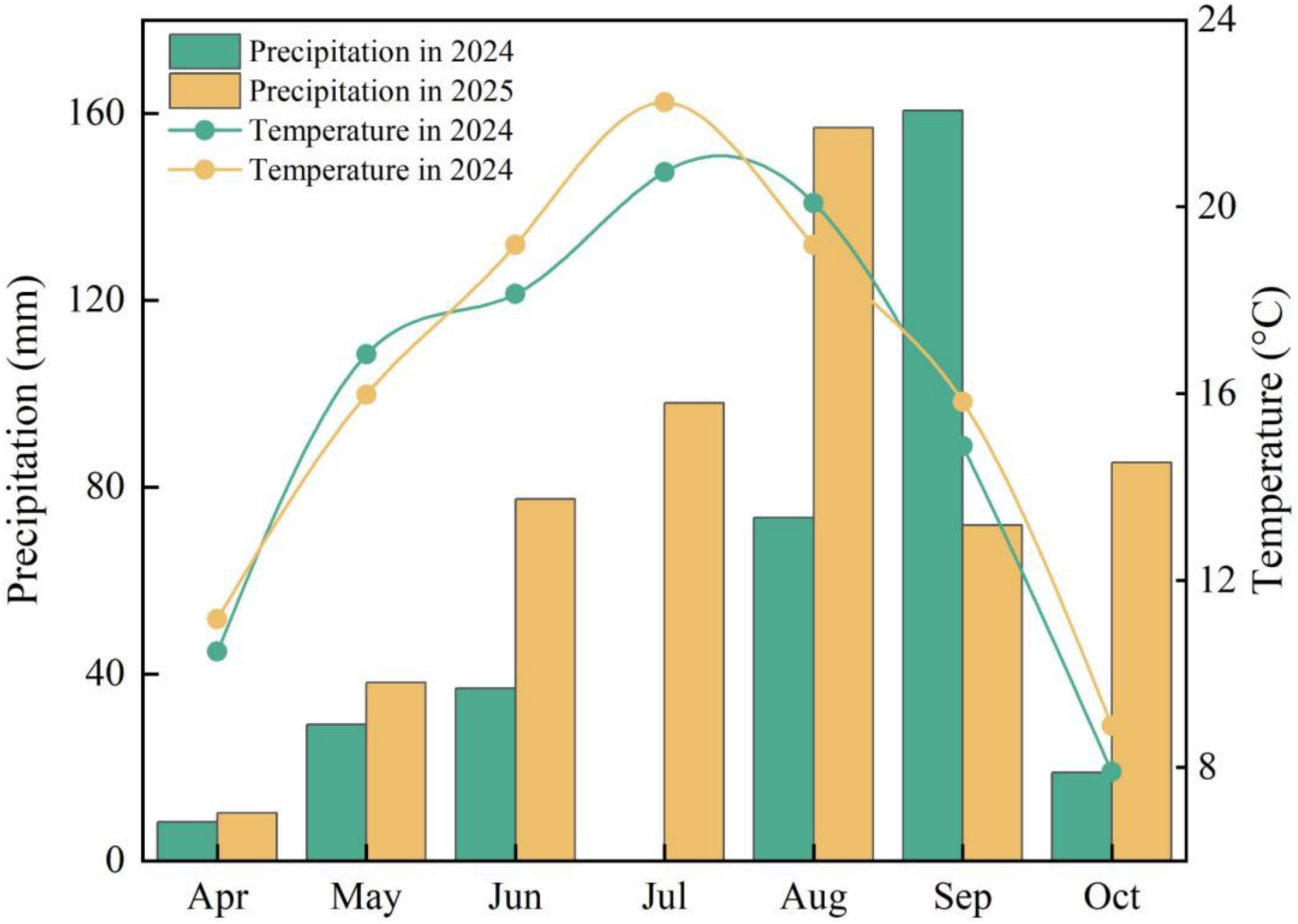
Monthly precipitation and air temperature in the study area from 2024 and 2025.

The field experiment was conducted during the alfalfa growing seasons from April to September in 2024 and 2025 at the Yinda Irrigation District Research Base of Gansu Agricultural University. The experimental site is located in Liaojiacao Village, Zhongchuan Town, Yongdeng County, Gansu Province, China (102°36′–103°45′ E, 36°12′–37°07′ N), and is characterized by a temperate continental climate. During 2024–2025, the mean annual air temperature was 15.9 °C, and the annual precipitation was 357.9 mm, approximately 70% of which occurred from June to September (**Figure 1**). The mean annual sunshine duration was 2659 h, and the frost-free period was approximately 121 d. The region is generally characterized by cold winters, hot summers, large diurnal temperature variation, low precipitation, and dry air conditions. The soil at the experimental site was sandy loam, with a bulk density of 1.37 g cm^-^³, a pH of 8.7, and a field capacity of 22.0% on a gravimetric water-content basis.

### 2.2 Experimental design

The experiment was conducted under drip irrigation. The alfalfa cultivar used in this study was “Jieyi”, provided by the Gansu Academy of Agricultural Sciences. Alfalfa was sown in mid-April 2023 using row planting, with a seeding rate of 30 kg ha^-1^ and a row spacing of 30 cm. A two-factor randomized block design was used, with irrigation level and phosphorus fertilization rate as the two experimental factors. Three irrigation levels were established according to soil field capacity: 45%–60%, 60%–75%, and 75%–90% of field capacity, designated as W1, W2, and W3, respectively (Ismail and Almarshadi, 2013). Three phosphorus fertilization rates were applied: 0, 50, and 100 kg ha^-1^ P_2_O_5_, designated as P0, P50, and P100, respectively (Zhang et al., 2020c). The combination of these two factors resulted in nine treatments: W1P0, W1P50, W1P100, W2P0, W2P50, W2P100, W3P0, W3P50, and W3P100. Each treatment was replicated three times, giving a total of 27 plots. Each plot measured 3 m × 6 m, with a 50 cm buffer zone between adjacent plots.

Irrigation was applied using in-line drip irrigation tapes laid along the alfalfa rows. The spacing between drip tapes was 25 cm, and the emitter spacing was 30 cm. The nominal diameter and wall thickness of the drip tapes were 16 mm and 0.2 mm, respectively, and the designed emitter discharge rate was 2.0 L h^-1^. Phosphorus fertilizer was applied by broadcasting rather than through fertigation. It was split into three applications during the growing season: the first at the regreening stage in early April, when the new alfalfa shoots reached approximately 5 cm in height; the second after the first cutting; and the third after the second cutting. This split application was used to meet the phosphorus demand of alfalfa during different growth periods. Before the experiment began, all plots received the same basal fertilization, consisting of potassium sulfate (K_2_O ≥ 50%) at 90 kg ha^-1^ and urea (N ≥ 46%) at 70 kg ha^-1^. Other field management practices were consistent with local conventional management. Based on the actual growth progress of alfalfa at the experimental site, the growth period of each cutting was divided into two main stages: branching to budding and budding to early flowering. Alfalfa was harvested at the early flowering stage for yield and quality measurements. For the first cutting, the branching-to-budding stage lasted from 15 April to 20 May, totaling 35 d, and the budding-to-early-flowering stage lasted from 21 May to 10 June, totaling 20 d. For the second cutting, the branching-to-budding stage lasted from 22 June to 14 July, totaling 22 d, and the budding-to-early-flowering stage lasted from 15 July to 29 July, totaling 14 d. For the third cutting, the branching-to-budding stage lasted from 12 August to 8 September, totaling 27 d, and the budding-to-early-flowering stage lasted from 9 September to 28 September, totaling 19 d. Water and phosphorus regulation was conducted at the branching and budding stages, and sampling was completed at the early flowering stage of each cutting, and the corresponding growth periods are provided in **Table 1**.

**Table 1:** Growth stages and corresponding periods of the three alfalfa cuttings during the experimental period.

Crop	Cutting	Growth stage	Period	Duration (d)
Alfalfa	First cutting	Branching to budding	Apr. 15–May 20	35
Alfalfa	First cutting	Budding to early flowering	May 21–Jun. 10	20
Alfalfa	Second cutting	Branching to budding	Jun. 22–Jul. 14	22
Alfalfa	Second cutting	Budding to early flowering	Jul. 15–Jul. 29	14
Alfalfa	Third cutting	Branching to budding	Aug. 12–Sep. 8	27
Alfalfa	Third cutting	Budding to early flowering	Sep. 9–Sep. 28	19

The irrigation treatments were established based on soil field capacity (FC). Three irrigation regimes were applied: W1, moderate water stress, targeted at 45%–60% of FC; W2, mild water stress, targeted at 60%–75% of FC; and W3, full irrigation, targeted at 75%–90% of FC. During the experimental period, soil water content was measured at 10–15 day intervals, Soil water content was monitored in three soil layers, namely 0–20, 20–40, and 40–60 cm, and the average soil water content of the 0–60 cm profile was used for irrigation scheduling, and irrigation was applied when soil water content decreased to the lower threshold of the corresponding irrigation treatment. The irrigation amount was measured using water meters and adjusted according to soil moisture status and meteorological conditions. Ridges were constructed around each plot to prevent surface runoff and water movement between plots. The irrigation frequency and amount for each cutting were determined according to the soil moisture control targets. During the first cutting, all irrigation treatments were irrigated three times, with total irrigation amounts of 501.0, 604.5, and 661.5 m³ ha^-1^ for W1, W2, and W3, respectively. During the second cutting, all treatments were irrigated three times, with total irrigation amounts of 493.5, 591.0, and 672.0 m³ ha^-1^, respectively. During the third cutting, all treatments were irrigated twice, with total irrigation amounts of 322.5, 385.5, and 436.5 m³ ha^-1^, respectively. Over the whole growing season, the cumulative irrigation amounts for W1, W2, and W3 were 1317.0, 1581.0, and 1770.0 m³ ha^-1^, respectively. The irrigation amount for each irrigation event is provided in **Supplementary Table 1**.

### 2.3 Measurement of leaf photosynthetic characteristics and SPAD value

During the alfalfa growing period, leaf photosynthetic characteristics were measured using a LI-6400XT portable photosynthesis system (LI-COR Biosciences, Lincoln, NE, USA). The measured parameters included net photosynthetic rate (*P*n), transpiration rate (*T*r), stomatal conductance (*G*s), and intercellular CO_2_ concentration (*C*i). Measurements were conducted on clear and windless days between 9:00 and 11:00. For each treatment, six healthy plants with uniform growth were randomly selected, and fully expanded, undamaged, and uniformly illuminated functional leaves located at approximately two-thirds of the plant height were used for measurement. Each leaf was measured five times, and the mean value was used as the representative value for that leaf. Relative chlorophyll content was determined using a SPAD-502 chlorophyll meter (Konica Minolta Sensing, Inc., Osaka, Japan).

Relative chlorophyll content was measured using a SPAD-502 Plus chlorophyll meter. Three points were selected on each fully expanded leaf, and the mean value was used as the SPAD value for each treatment. Instantaneous water use efficiency (WUEi) was calculated as follows:


$$WUE_{i}=\frac{Pn}{Tr}$$

### 2.4 Measurement of plant morphological traits

Considering the regrowth characteristics of alfalfa, a multiple-cutting harvest system was used. Based on previous studies on alfalfa cultivation and harvest management, the early flowering stage was selected as the harvest time to balance yield and forage quality (Ma, 2018). At each cutting, alfalfa was harvested at the early flowering stage. Four 1 m × 1 m quadrats were randomly selected from each plot, and plants were cut at a stubble height of 5 cm. Fresh weight was measured immediately after harvest. The samples were then oven-dried at 105 °C for 30 min to deactivate enzymes and subsequently dried at 75 °C to constant weight. Dry weight was recorded, and the mean value was used to calculate hay yield per unit area, expressed as kg ha^-1^.

Before each cutting, healthy plants with uniform growth and no visible pest or disease damage were randomly selected from each plot for morphological measurements. Five plants were randomly selected from each plot. Plant height was measured from the soil surface to the plant apex using a measuring tape and expressed in cm. Stem diameter was measured 5 cm above the root crown using a vernier caliper and recorded to an accuracy of 0.01 mm. The number of branches per plant was counted, and the mean value was used as the branch number for each plot. For leaf-to-stem ratio determination, ten healthy and uniformly growing plants were randomly selected from each plot and cut at 5 cm above the soil surface. Leaves were manually separated from stems and branches and placed into paper envelopes. The fresh weight of each component was recorded. Samples were then oven-dried at 105 °C for 30 min and subsequently dried at 75 °C to constant weight.

### 2.5 Measurement of hay yield and forage quality

Forage quality was measured using samples collected simultaneously with yield determination. For each treatment, three representative fresh forage samples were randomly collected, with each sample weighing 10 g. Samples were placed in sealed bags and transported to the laboratory. They were first oven-dried at 105 °C for 30 min and then dried at 75 °C to constant weight. The dried samples were ground using a stainless-steel grinder and passed through a 1 mm sieve for subsequent quality analyses. Crude protein (CP) content was determined using the Kjeldahl method. Crude ash content was determined by dry ashing. Neutral detergent fiber (NDF) and acid detergent fiber (ADF) were determined using the Van Soest method.

### 2.6 Measurement of soil available nutrients

Before each alfalfa cutting, soil samples were collected from each treatment plot using an “S-shaped” sampling method. Three sampling points were randomly selected in each plot, and soil samples were collected from the 0–20, 20–40, and 40–60 cm soil layers. Plant roots, stones, and other debris were removed, and soil samples from the same depth within each plot were thoroughly mixed and transported to the laboratory for analysis.

Soil ammonium nitrogen (NH_4_^+^-N), nitrate nitrogen (NO_3_^-^-N), available phosphorus (AP), and available potassium (AK) were determined using a Liande LD-GT5 soil nutrient analyzer.

### 2.7 Calculation of irrigation water use efficiency and phosphorus agronomic efficiency

Irrigation water use efficiency (IWUE) was calculated as follows:


$$IWUE=\frac{Y}{I}$$

where *Y* is alfalfa hay yield (kg ha^-1^), and *I* is the irrigation amount during the corresponding growth period.

Phosphorus agronomic efficiency (AEP) was used to quantify the yield increase per unit phosphorus fertilizer input and was calculated as follows:


$$AEP=\frac{Y_{P}-Y_{0}}{F_{P}}$$

where *Y_P_* is the yield under phosphorus fertilization, *Y*_0_ is the yield under no phosphorus application, and *F_P_* is the phosphorus fertilizer application rate. AEP was expressed as kg kg^-1^ P_2_O_5_.

### 2.8 Construction of comprehensive indices

The comprehensive photosynthetic index (CPI) was used to evaluate the overall photosynthetic performance of alfalfa under different treatments. Net photosynthetic rate (Pn), transpiration rate (Tr), stomatal conductance (Gs), intercellular CO_2_ concentration (Ci), instantaneous water use efficiency (WUEi), and SPAD value were first normalized using min–max normalization to eliminate differences in units and scales. CPI was then calculated using the normalized values and the weighting coefficients derived from PCA:


$$X_{i}^{\prime }=\frac{X_{i}-X_{min}}{X_{max}-X_{min}}\;CPI=\sum_{i=1}^{n}W_{i}X_{i}^{\prime }$$

where $X_{i}^{\prime }$ represents the normalized value of the *i*th photosynthetic parameter, and *W_i_* represents the normalized PCA-derived weight of that parameter.

The forage quality index (FQI) was used to evaluate the overall nutritive value of forage under different treatments. Crude protein (CP), crude ash (Ash), neutral detergent fiber (NDF), and acid detergent fiber (ADF) were normalized using min–max normalization. CP and Ash were treated as positive indicators, whereas NDF and ADF were treated as negative indicators. Weighting coefficients were then derived from PCA loadings and normalized. A higher FQI value indicates better overall forage quality.


$$FQI=\sum_{i=1}^{n}W_{i}X_{i}^{\prime }$$

where $X_{i}^{\prime }$ represents the normalized value of each forage quality indicator, and *W_i_* represents the corresponding normalized weight.

The comprehensive morphological index (CMI) was used to evaluate plant morphological development and overall growth status under different treatments. Plant height (PH), stem diameter (SD), branch number (BN), and stem-to-leaf ratio (SLR) were selected as morphological indicators. PH, SD, and BN were treated as positive indicators because higher values generally indicate stronger plant growth vigor, whereas SLR was treated as a negative indicator because a higher stem-to-leaf ratio usually reflects a lower leaf proportion and poorer forage quality.

For positive indicators, normalization was performed as follows:


$$X_{i}^{\prime }=\frac{X_{i}-X_{min}}{X_{max}-X_{min}}$$

For the negative indicator, normalization was performed as follows:


$$X_{i}^{\prime }=\frac{X_{max}-X_{i}}{X_{max}-X_{min}}$$

The CMI was calculated as follows:


$$CMI=\frac{PH^{\prime }+SD^{\prime }+BN^{\prime }+SLR^{\prime }}{4}$$

where *PH*′, *SD*′, *BN*′, and *SLR*′ represent the normalized values of plant height, stem diameter, branch number, and stem-to-leaf ratio, respectively. A higher CMI value indicates better morphological development and overall plant growth performance. The PCA-derived weighting coefficients for each indicator used in the calculation of CPI, FQI, and CMI are provided in **Supplementary Table 2**.

The soil quality index was calculated based on soil available nutrient indicators in the 0–60 cm soil profile. First, soil properties measured at 0–20, 20–40, and 40–60 cm were integrated into a single value for the 0–60 cm profile using a depth-weighted average. Because the three soil layers had the same thickness, the calculation was as follows:


$$X_{0-60}=\frac{X_{0-20}+X_{20-40}+X_{40-60}}{3}$$

All soil indicators were then normalized to eliminate differences in units and scales. For positive indicators, the following min–max normalization method was used:


$$S_{i}=\frac{X_{i}-X_{\text{min}}}{X_{\text{max}}-X_{\text{min}}}$$

The soil quality index was calculated as follows:


$$SQI=\sum_{i=1}^{n}W_{i}\times S_{i}$$

where *SQI* represents the soil quality index, *S_i_* is the normalized value of the *i* the soil indicator, *W_i_* is the weight of the corresponding indicator, and *n* is the number of soil indicators included in the index. The weights were determined using principal component analysis, ensuring methodological consistency with the calculation of CPI, FQI, and CMI.

### 2.9 Entropy-weight TOPSIS evaluation

To identify the optimal irrigation and phosphorus fertilization combination that balanced multiple benefits, an entropy-weighted TOPSIS method was used for comprehensive evaluation. Seven indicators, including forage yield, IWUE, AEP, CPI, FQI, CMI, and soil quality index (SQI), were included in the evaluation system. All indicators were treated as benefit-type indicators, with higher values indicating better performance. Since AEP is not applicable to non-phosphorus treatments, the AEP values of P0 treatments were set to zero in the comprehensive evaluation. The original data matrix was first normalized using the min–max normalization method. The entropy method was then used to calculate the objective weight of each indicator. Based on the weighted normalized matrix, the distances of each treatment from the positive and negative ideal solutions were calculated. Finally, the closeness coefficient was obtained to rank the comprehensive performance of different treatments. Each replicate was used as an independent evaluation unit, and the average TOPSIS score of the three replicates was used to determine the final ranking of each treatment. A higher closeness coefficient indicated better comprehensive performance.

Let $X={\left(x_{ij}\right)}_{m\times n}$ be the original data matrix, where *m* is the number of treatments and *n* is the number of evaluation indicators. The indicators were first normalized as follows:

For positive indicators:


$$z_{ij}=\frac{x_{ij}-\text{min}\left(x_{j}\right)}{\text{max}\left(x_{j}\right)-\text{min}\left(x_{j}\right)}$$

For negative indicators:


$$z_{ij}=\frac{\text{max}\left(x_{j}\right)-x_{ij}}{\text{max}\left(x_{j}\right)-\text{min}\left(x_{j}\right)}$$

The proportion of the *i*-th treatment under the *j*-th indicator was calculated as:


$$p_{ij}=\frac{z_{ij}}{\sum_{i=1}^{m}z_{ij}}$$

The entropy value of each indicator was calculated as:


$$e_{j}=-k\sum_{i=1}^{m}p_{ij}\text{ln}\left(p_{ij}\right),k=\ln m$$

The information redundancy and entropy weight were calculated as:


$$d_{j}=1-e_{j}\;w_{j}=\frac{d_{j}}{\sum_{j=1}^{n}d_{j}}$$

The weighted normalized matrix was then obtained:


$$v_{ij}=w_{j}z_{ij}$$

The positive and negative ideal solutions were defined as:


$$V_{j}^{+}=\text{max}\left(v_{ij}\right),V_{j}^{-}=\text{min}\left(v_{ij}\right)$$

The distances from each treatment to the positive and negative ideal solutions were calculated as:


$$D_{i}^{+}=\sqrt{\sum_{j=1}^{n}{\left(v_{ij}-V_{j}^{+}\right)}^{2}}\;D_{i}^{-}=\sqrt{\sum_{j=1}^{n}{\left(v_{ij}-V_{j}^{-}\right)}^{2}}$$

Finally, the TOPSIS closeness coefficient was calculated as:


$$C_{i}=\frac{D_{i}^{-}}{D_{i}^{+}+D_{i}^{-}}$$

where *C_i_* represents the comprehensive evaluation score of each treatment. A higher *C_i_* value indicates better overall performance.

### 2.10 Statistical analysis

Raw data were first organized using Microsoft Excel. Statistical analyses were performed using SPSS 24.0. All measured variables were expressed as mean ± standard deviation (mean ± SD). Multifactor analysis of variance (ANOVA) was used to test the effects of irrigation level, phosphorus fertilization rate, year, and their interactions on alfalfa yield, forage quality, photosynthetic characteristics, and soil available nutrients. In addition, one-way ANOVA was used to analyze the significance of differences among different water–phosphorus treatment combinations. Principal component analysis (PCA) was performed using SPSS 24.0 to construct comprehensive evaluation indices and assess the integrated effects of different treatments. All figures were generated using Origin 2024b.

## 3 Results

### 3.1 Effects of year, irrigation, and phosphorus fertilization on measured indicators

As shown in **Table 2**, significant interannual differences were observed for several measured indicators. Compared with 2024, the 2025 values were significantly higher for net photosynthetic rate (*P*_n_), transpiration rate (*T*r), stomatal conductance (*G*s), instantaneous water use efficiency (WUE_i_), SPAD value, neutral detergent fiber (NDF), acid detergent fiber (ADF), plant height (PH), stem diameter (SD), branch number (BN), ammonium nitrogen (NH_4_^+^-N), irrigation water use efficiency (IWUE), agronomic phosphorus efficiency (AEP), and yield, with Year effects ranging from 4.3* to 346***. By contrast, intercellular CO_2_ concentration (CI), comprehensive photosynthetic index (CPI), crude protein (CP), crude ash (CA), forage quality index (FQI), leaf-to-stem ratio (LSR), comprehensive morphological index (CMI), nitrate nitrogen (NO_3_^-^-N), available phosphorus (AP), available potassium (AK), and soil quality index (SQI) showed no significant differences between 2024 and 2025.

**Table 2:** Interannual variation in alfalfa growth, physiological, quality, soil, and productivity indicators across 2024 and 2025.

Indicator	2024	2025	Year effect
Pn	19.7B	20.5A	346***
Tr	11.0B	11.2A	81.0***
Gs	0.7B	0.7A	91.4***
Ci	334A	333A	ns
WUEi	1.8B	1.8A	108***
SPAD	60.5B	62.1A	27.6***
CPI	0.5A	0.5A	ns
CP	13.3A	13.2A	ns
CA	13.1A	13.1A	ns
NDF	40.4B	41.0A	264***
ADF	26.1B	26.4A	179***
FQI	0.5A	0.5A	ns
PH	86.5B	88.4A	5.2*
SD	3.6B	3.7A	43.3***
LSR	0.8A	0.8A	ns
BN	14.9B	15.2A	7.3*
CMI	0.5A	0.5A	ns
NH_4_^+^-N	67.8B	69.8A	21.2***
NO_3_^-^-N	70.4A	69.8A	ns
AP	26.9A	27.0A	ns
AK	158A	159A	ns
SQI	0.4A	0.4A	ns
IWUE	4.6B	4.8A	269***
AEP	8.6B	10.4A	4.3*
Yield	21161B	22174A	288***

* indicates significance at P < 0.05, and *** indicates significance at P < 0.001.

According to the three-way ANOVA results, year (Y), irrigation (W), phosphorus fertilization (P), and their interactions had variable effects on the measured indicators (**Table 3**). Year significantly affected *P*_n_, *T*_r_, *G*_s_, *C*_i_, SPAD, NDF, ADF, PH, SD, BN, IWUE, AEP, and yield, whereas its effects on *C*_i_, CPI, CP, CA, FQI, LSR, CMI, and SQI were not significant. Irrigation significantly affected all measured indicators, with relatively high F values observed for *P*_n_, CPI, IWUE, ADF, *T*_r_, *G*_s_, and WUEi. Phosphorus fertilization significantly affected *P*_n_, *T*_r_, *G*_s_, *C*_i_, WUE_i_, SPAD, CPI, CP, CA, NDF, ADF, FQI, PH, BN, CMI, IWUE, AEP, and yield, but had no significant effect on SD, LSR, and SQI. For the interaction terms, Y × W significantly affected *P*_n_, *T*_r_, NDF, ADF, PH, SD, IWUE, AEP, and yield. The Y × P interaction was significant for NDF, PH, LSR, BN, CMI, and AEP. The W × P interaction significantly affected *P*_n_, WUE_i_, SPAD, CPI, CP, CA, NDF, ADF, FQI, IWUE, AEP, and yield. Among all measured indicators, only AEP showed a significant Y × W × P interaction, while no significant three-way interaction was detected for the remaining indicators.

**Table 3:** Three-way ANOVA of year, irrigation, and phosphorus fertilization effects on measured alfalfa and soil indices.

Indicator	Y	W	P	Y×W	Y×P	W×P	Y×W×P
Pn	346***	1820***	104***	6.7**	ns	26.1***	ns
Tr	81.0***	658***	5.7**	3.9*	ns	ns	ns
Gs	91.4***	576***	9.1***	ns	ns	ns	ns
Ci	ns	99.2***	4.2*	ns	ns	ns	ns
WUEi	108***	534***	64.6***	ns	ns	20.0***	ns
SPAD	27.6***	52.4***	27.3***	ns	ns	13.3***	ns
CPI	ns	1370***	89.6***	ns	ns	18.9***	ns
CP	ns	76.7***	33.7***	ns	ns	5.1**	ns
CA	ns	159***	21.3***	ns	ns	13.0***	ns
NDF	264***	153***	52.7***	13.0***	3.6*	10.2***	ns
ADF	179***	802***	163***	13.6***	ns	42.2***	ns
FQI	ns	47.8***	3.3*	ns	ns	3.4*	ns
PH	5.2*	12.4***	5.0*	4.2*	5.2*	ns	ns
SD	43.3***	164***	ns	3.4*	ns	ns	ns
LSR	ns	19.8***	ns	ns	4.1*	ns	ns
BN	7.3*	7.4**	7.0**	ns	7.0**	ns	ns
CMI	ns	44.8***	6.1**	ns	6.4**	ns	ns
NH_4_^+^-N	***	***	ns	ns	ns	ns	ns
NO_3_^-^-N	ns	***	ns	ns	ns	ns	ns
AP	ns	***	*	ns	ns	***	ns
AK	ns	***	**	ns	ns	ns	ns
SQI	ns	190***	ns	ns	ns	ns	ns
IWUE	269***	2235***	106***	12.4***	ns	4.1**	ns
AEP	4.3*	10.2***	145***	5.8**	3.7*	6.7***	3.9**
Yield	288***	167***	120***	6.3**	ns	4.6**	ns

* indicates significance at P < 0.05, and *** indicates significance at P < 0.001.

### 3.2 Effects of water–phosphorus coupling on alfalfa yield and irrigation water use efficiency

**Figure 2:**
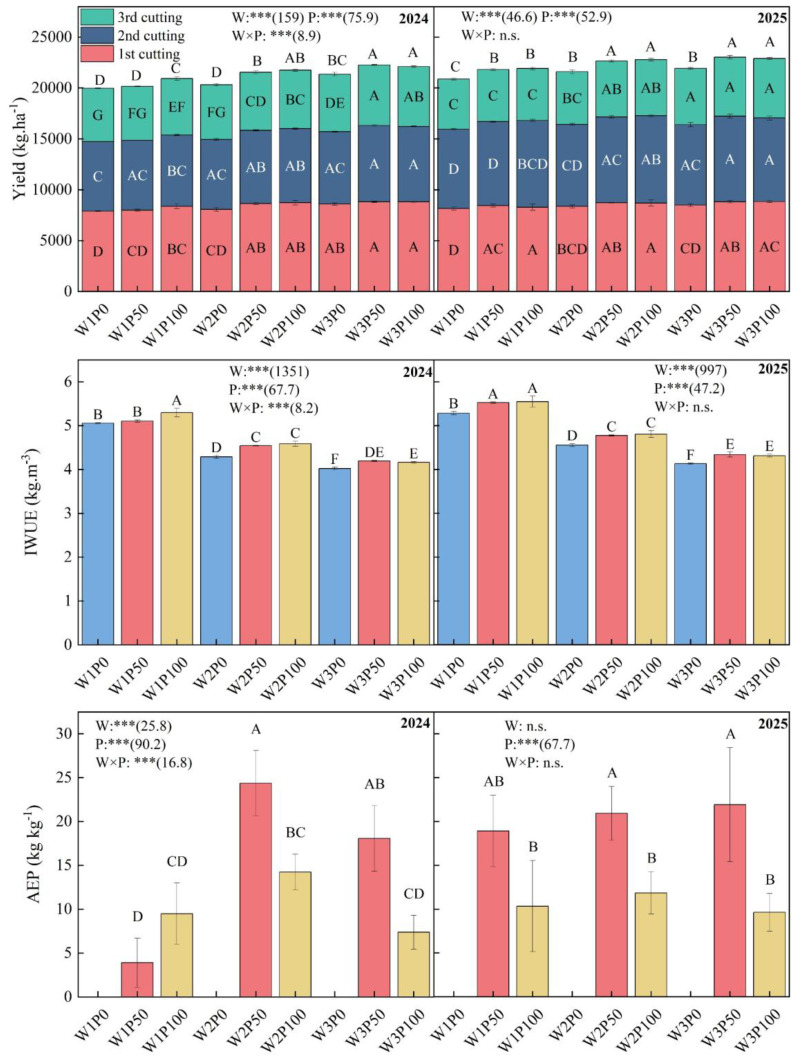
Effects of different irrigation and phosphorus fertilization combinations on alfalfa yield, irrigation water use efficiency (IWUE) and phosphorus agronomic efficiency (AEP). W1, W2, and W3 indicate moderate water stress, mild water stress, and full irrigation, respectively. P0, P50, and P100 indicate phosphorus application rates of 0, 50, and 100 kg ha^-2^, respectively. Different uppercase letters indicate significant differences among treatments at (*P<* 0.05). W, and P represent the significance of irrigation and phosphorus fertilization respectively. *, **, ***, and n.s. indicate p< 0.05, p< 0.01, p< 0.001, and p > 0.05, respectively. Values in parentheses indicate F-values.

Irrigation and phosphorus fertilization significantly affected alfalfa yield, irrigation water use efficiency (IWUE), and agronomic phosphorus efficiency (AEP), while their interaction was significant only in 2024 (P< 0.05; **Figure 2**). After the first cutting in 2024, the yields under W2P50 and W3P50 were significantly higher than that under W1P50, increasing by 8.2% and 10.4%, respectively, with no significant difference between W2P50 and W3P50. In 2025, the yields under W2P50 and W3P50 increased by 3.3% and 4.4%, respectively, compared with W1P50. For the second and third cuttings in both 2024 and 2025, W2P50 and W3P50 consistently produced significantly higher yields than W1P50. Annual yield under W2P50 and W3P50 was significantly higher than that under W1P50, while annual yield under W2P100 and W3P100 was significantly higher than that under W1P100. In 2025, no significant differences were observed between W2P50 and W3P50 or between W2P100 and W3P100. IWUE under W2P50, W3P50, W2P100, and W3P100 was significantly lower than that under W1P50 and W1P100. In both years, AEP under W2P50 and W3P50 was significantly higher than that under W2P100 and W3P100; however, AEP under W2P50 and W3P50 was significantly higher than that under W1P50 only in 2024 (P< 0.05).

### 3.3 Effects of water–phosphorus coupling on alfalfa plant traits

**Figure 3:**
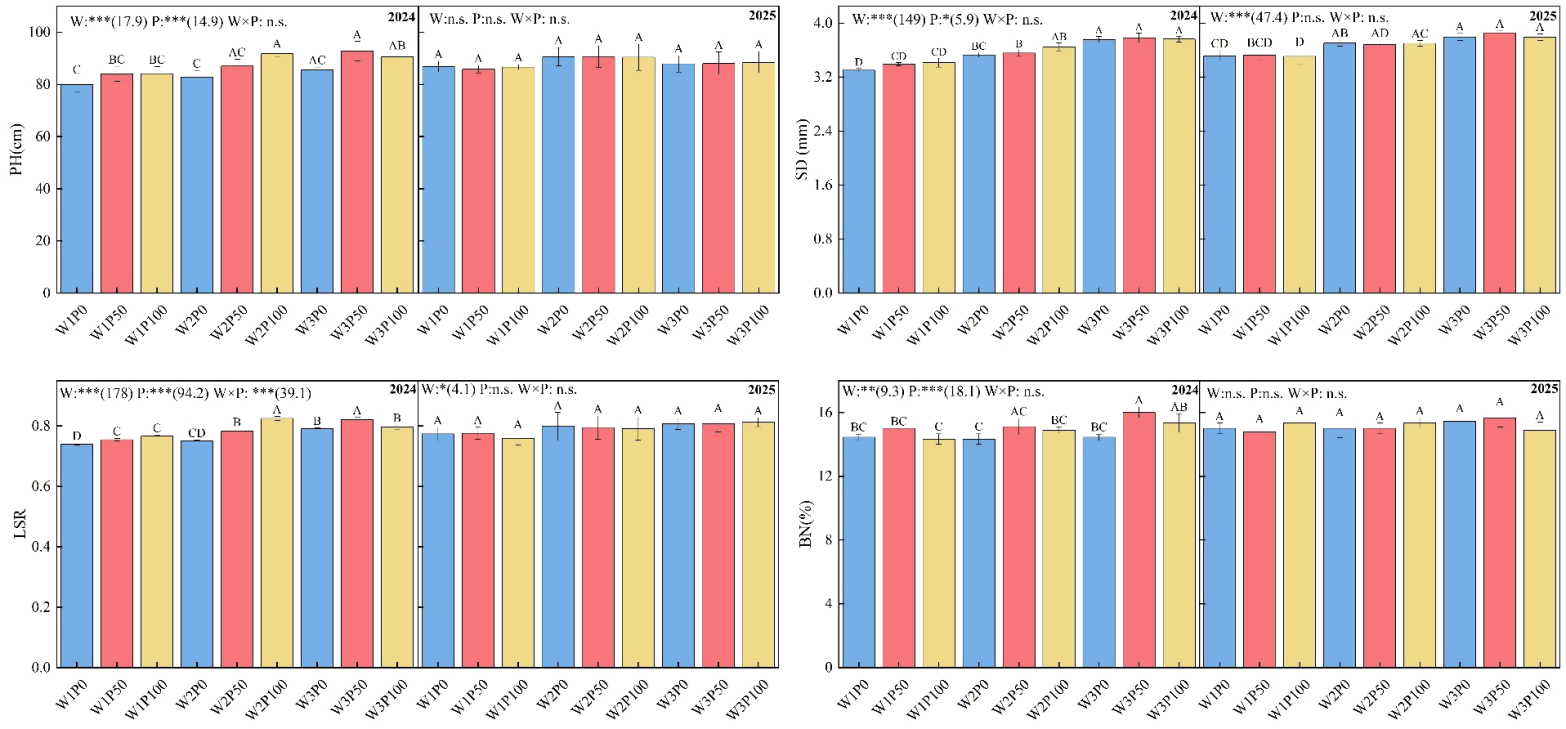
Effects of different irrigation and phosphorus fertilization combinations on alfalfa plant height (PH), stem diameter (SD), leaf-to-stem ratio (LSR), and branch number (BN). W1, W2, and W3 indicate moderate water stress, mild water stress, and full irrigation, respectively. P0, P50, and P100 indicate phosphorus application rates of 0, 50, and 100 kg ha^-2^, respectively. Different uppercase letters indicate significant differences among treatments at (P< 0.05). W, and P represent the significance of irrigation and phosphorus fertilization respectively. *, **, ***, and n.s. indicate p< 0.05, p< 0.01, p< 0.001, and p > 0.05, respectively. Values in parentheses indicate F-values.

Irrigation significantly affected plant height (PH), stem diameter (SD), leaf-to-stem ratio (LSR), and branch number (BN), while the interaction between irrigation and phosphorus fertilization significantly affected only LSR in 2024 (P< 0.05; **Figure 3**), except for PH and BN in 2025. In both 2024 and 2025, SD under W2P50 and W3P50, as well as under W2P100 and W3P100, was significantly higher than that under W1P50 and W1P100, respectively. In 2024, LSR under W2P50 and W3P50 was significantly higher than that under W1P50, and a significant difference was also observed between W2P50 and W3P50. In addition, PH under W2P50 and W3P50 was significantly higher than that under W1P50 in 2024, with no significant difference between W2P50 and W3P50.

### 3.4 Effects of water–phosphorus coupling on alfalfa forage quality

**Figure 4:**
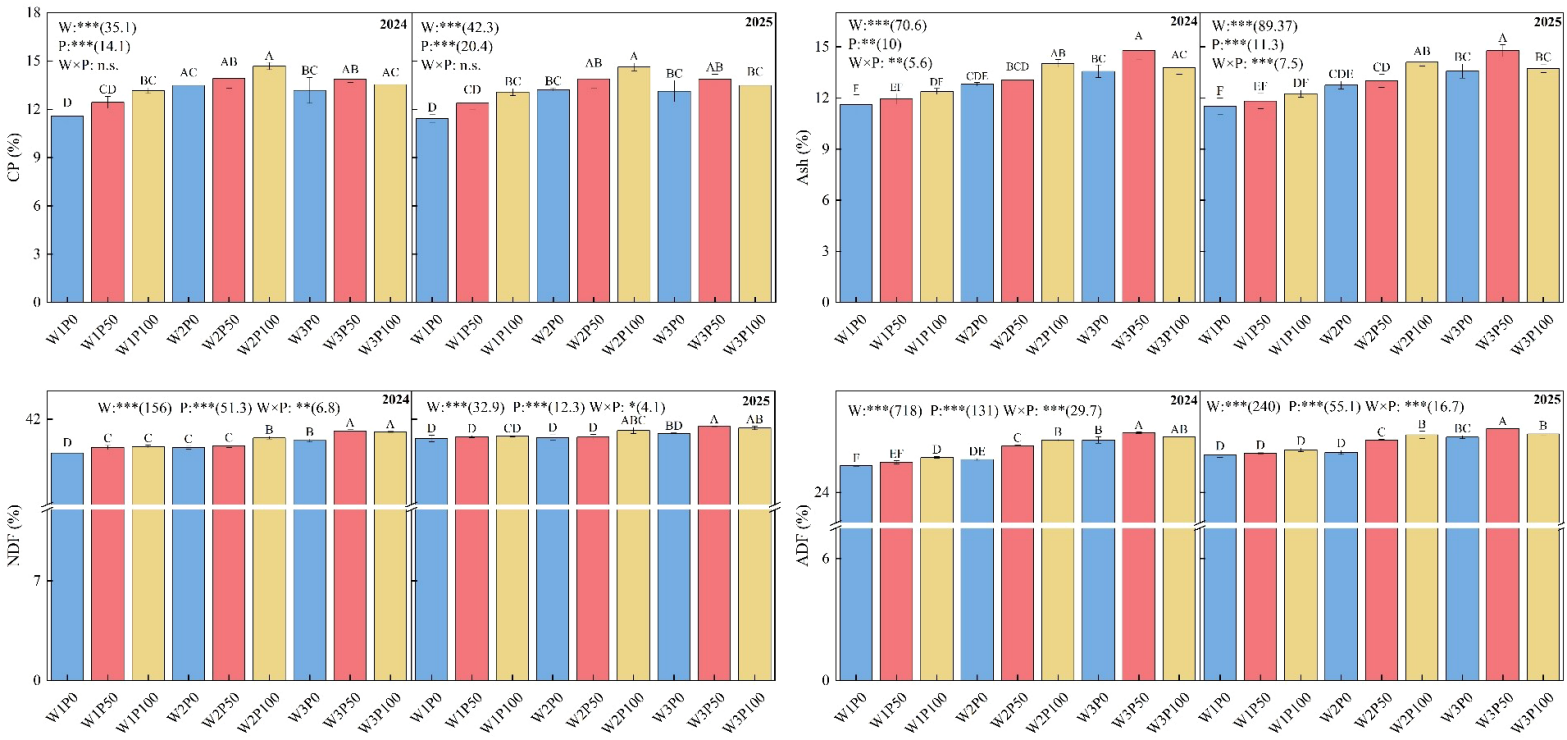
Effects of different irrigation and phosphorus fertilization combinations on crude protein (CP), crude ash (Ash), neutral detergent fiber (NDF), and acid detergent fiber (ADF) contents of alfalfa. W1, W2, and W3 indicate moderate water stress, mild water stress, and full irrigation, respectively. P0, P50, and P100 indicate phosphorus application rates of 0, 50, and 100 kg ha^-2^, respectively. Different uppercase letters indicate significant differences among treatments at (P< 0.05). W, and P represent the significance of irrigation and phosphorus fertilization respectively. *, **, ***, and n.s. indicate p< 0.05, p< 0.01, p< 0.001, and p > 0.05, respectively. Values in parentheses indicate F-values.

Irrigation and phosphorus fertilization significantly affected crude protein (CP), ash, neutral detergent fiber (NDF), and acid detergent fiber (ADF), while their interaction significantly affected only ash, NDF, and ADF (P< 0.05; **Figure 4**). CP under W2P50 and W3P50 was significantly higher than that under W1P50. Compared with W1P50, CP under W2P50 and W3P50 increased by 11.9% and 11.5% in 2024, and by 12.1% and 11.9% in 2025, respectively. In addition, compared with W1P50, only W2P50 significantly increased ADF and ash, whereas no significant difference was observed in NDF.

### 3.5 Effects of water–phosphorus coupling on photosynthetic characteristics

**Figure 5:**
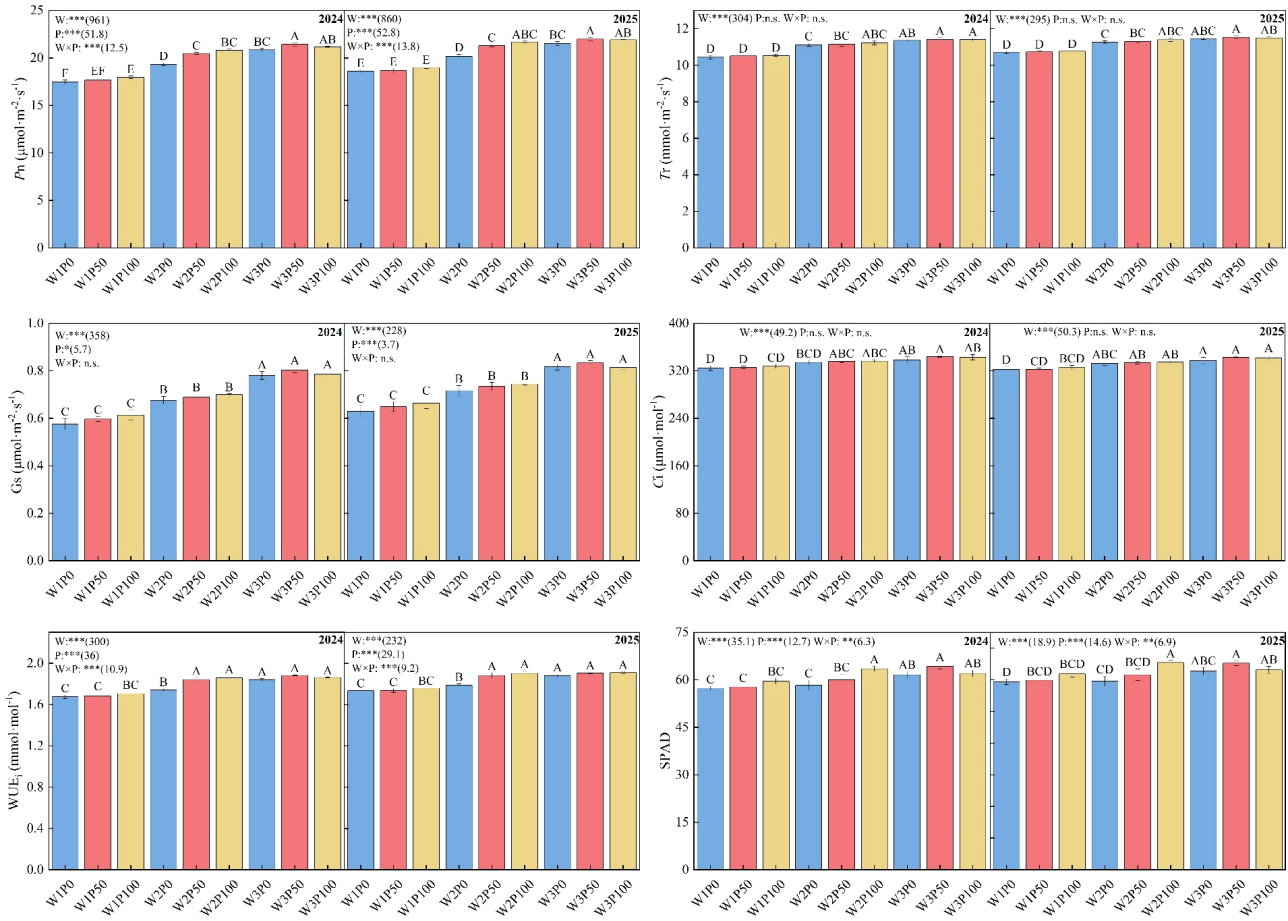
Effects of different irrigation and phosphorus fertilization combinations on net photosynthetic rate (*P*n), transpiration rate (*T*r), stomatal conductance (*G*s), intercellular CO_2_ concentration (*C*i), instantaneous water use efficiency (WUEi), and chlorophyll content (SPAD) of alfalfa leaves. W1, W2, and W3 indicate moderate water stress, mild water stress, and full irrigation, respectively. P0, P50, and P100 indicate phosphorus application rates of 0, 50, and 100 kg ha^-2^, respectively. Different uppercase letters indicate significant differences among treatments at (P< 0.05). W, and P represent the significance of irrigation and phosphorus fertilization respectively. *, **, ***, and n.s. indicate p< 0.05, p< 0.01, p< 0.001, and p > 0.05, respectively. Values in parentheses indicate F-values.

Irrigation and phosphorus fertilization significantly affected net photosynthetic rate (*P*n), stomatal conductance (*G*s), intrinsic water-use efficiency (WUEi), and SPAD value, while their interaction significantly affected only Pn, WUEi, and SPAD (P< 0.05; **Figure 5**). Under the same phosphorus level, *P*n, transpiration rate (*T*r), and Gs under W3 were significantly higher than those under W1 and W2 in both 2024 and 2025, and no significant difference in *P*n was observed between W3P50 and W3P100. In both years, WUE_i_ under W2P50 and W3P50 was significantly higher than that under W1P50, with no significant difference between W2P50 and W3P50. In addition, SPAD under W3P50 was significantly higher than that under W2P50 and W1P50, but did not differ significantly from that under W2P100 (P< 0.05).

### 3.6 Principal component analysis and comprehensive evaluation of photosynthesis, forage quality, and plant morphology

Across irrigation and phosphorus treatments, yield was highest under W3P50, with no significant difference from W3P100. Under the P50 level, yield under W2P50 and W3P50 increased by 5.3% and 7.9%, respectively, compared with W1P50. Under the P100 level, yield under W2P100 and W3P100 increased by 4.0% and 5.0%, respectively, compared with W1P100. Under W2 and W3 irrigation regimes, IWUE under P50 and P100 was significantly higher than that under P0, whereas no significant difference was observed between P50 and P100. AEP under W2P50 and W3P50 was significantly higher than that under W1P50 and W3P100, with no significant difference between W2P50 and W3P50. Soil quality index (SQI) under phosphorus fertilization in the W3 treatment was significantly higher than that under W2 and W1, and the interaction between irrigation and phosphorus fertilization significantly affected SQI.

### 3.7 Effects of water–phosphorus coupling on soil available nutrients

**Figure 6:**
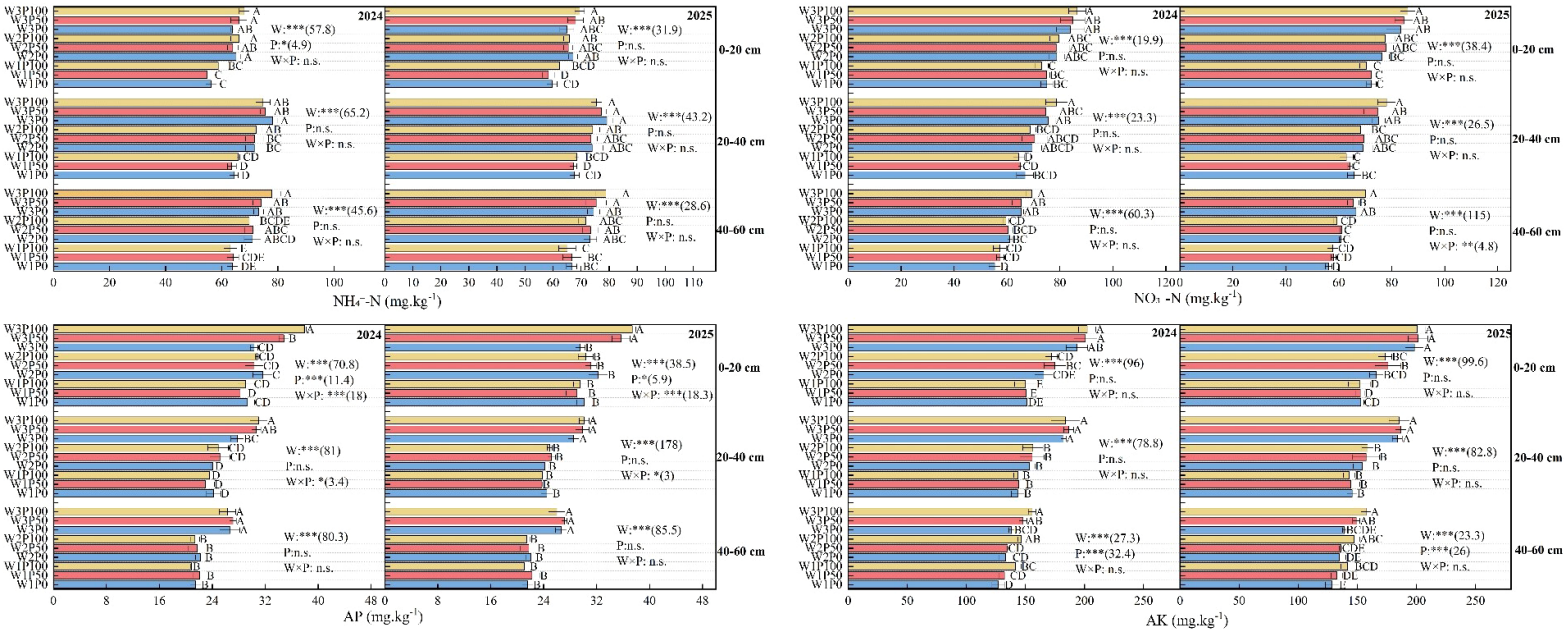
Effects of different irrigation and phosphorus fertilization combinations on soil ammonium nitrogen (NH_4_^+^-N), nitrate nitrogen (NO_3_^-^-N), available phosphorus (AP), and available potassium (AK) in the 0–20, 20–40, and 40–60 cm soil layers. W1, W2, and W3 indicate moderate water stress, mild water stress, and full irrigation, respectively. P0, P50, and P100 indicate phosphorus application rates of 0, 50, and 100 kg ha^-2^, respectively. Different uppercase letters indicate significant differences among treatments at (P< 0.05). W, and P represent the significance of irrigation and phosphorus fertilization respectively. *, **, ***, and n.s. indicate p< 0.05, p< 0.01, p< 0.001, and p > 0.05, respectively. Values in parentheses indicate F-values.

Irrigation significantly affected soil ammonium nitrogen (NH_4_^+^-N), nitrate nitrogen (NO_3_^-^-N), available phosphorus (AP), and available potassium (AK) in the 0–20, 20–40, and 40–60 cm soil layers (p< 0.05; **Figure 6**). Phosphorus fertilization significantly affected AP in all three soil layers. The interaction between irrigation and phosphorus fertilization significantly affected NH_4_^+^-N only in the 0–20 cm soil layer, and AP only in the 0–20 and 20–40 cm soil layers. NH_4_^+^-N did not differ significantly among irrigation and phosphorus treatments across the different soil layers. However, NO_3_^-^-N, AP, and AK under W2P50, W2P100, W3P50, and W3P100 were generally higher than those under W1 combined with phosphorus fertilization. The highest NO_3_^-^-N, AP, and AK values were generally observed under W3P50 and W3P100, with no significant difference between these two treatments.

**Figure 7:**
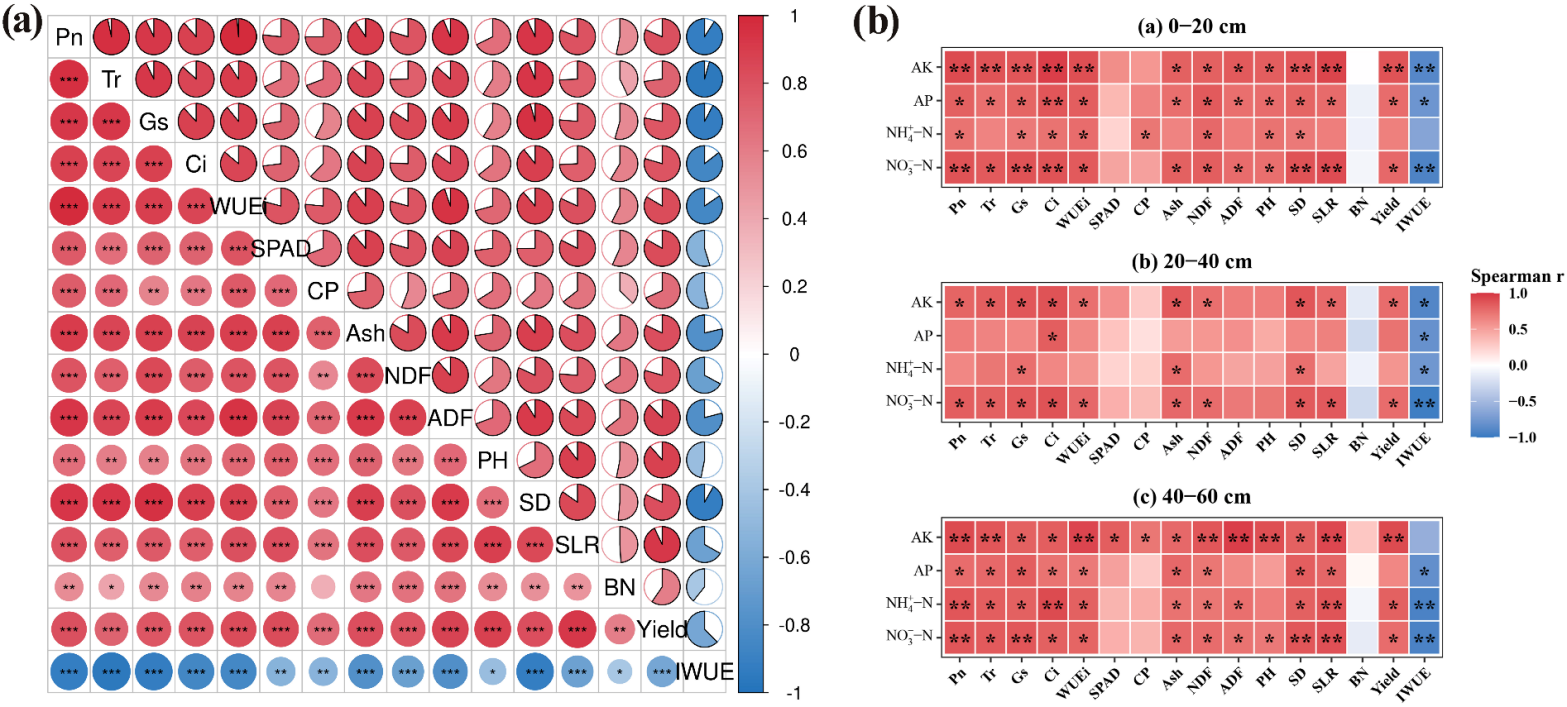
Correlation analysis among alfalfa photosynthetic characteristics, forage quality, plant morphological traits, yield, and irrigation water use efficiency **(A)**, and correlations of soil ammonium nitrogen (NH_4_^+^-N), nitrate nitrogen (NO_3_^-^-N), available phosphorus (AP), and available potassium **(AK)** in the 0–20, 20–40, and 40–60 cm soil layers with alfalfa photosynthetic characteristics, forage quality, plant morphological traits, yield, and irrigation water use efficiency **(B)**. *, **, and *** indicate significance at P < 0.05, P < 0.01, and P < 0.001, respectively.

Alfalfa yield was significantly positively correlated with photosynthetic characteristics, forage quality traits, and plant morphological traits, whereas IWUE was significantly negatively correlated with these indicators (p< 0.05; **Figure 7**). In the 0–20 cm soil layer, soil NO_3_^-^-N, AP, and AK were significantly positively correlated with *P*n, *T*r, *G*s, *C*i, WUE_i_, NDF, ADF, Ash, PH, SD, LSR, and yield. In the 20–40 cm soil layer, soil nutrients were significantly positively correlated with Ci, Ash, and SD. In the 40–60 cm soil layer, NO_3_^-^-N, AP, and AK were significantly positively correlated with *P*n, *T*r, *G*s, *C*i, WUEi, NDF, ADF, Ash, SD, LSR, and yield. Across the 0–20, 20–40, and 40–60 cm soil layers, NH_4_^+^-N was significantly positively correlated with *G*s and SD.

**Figure 8:**
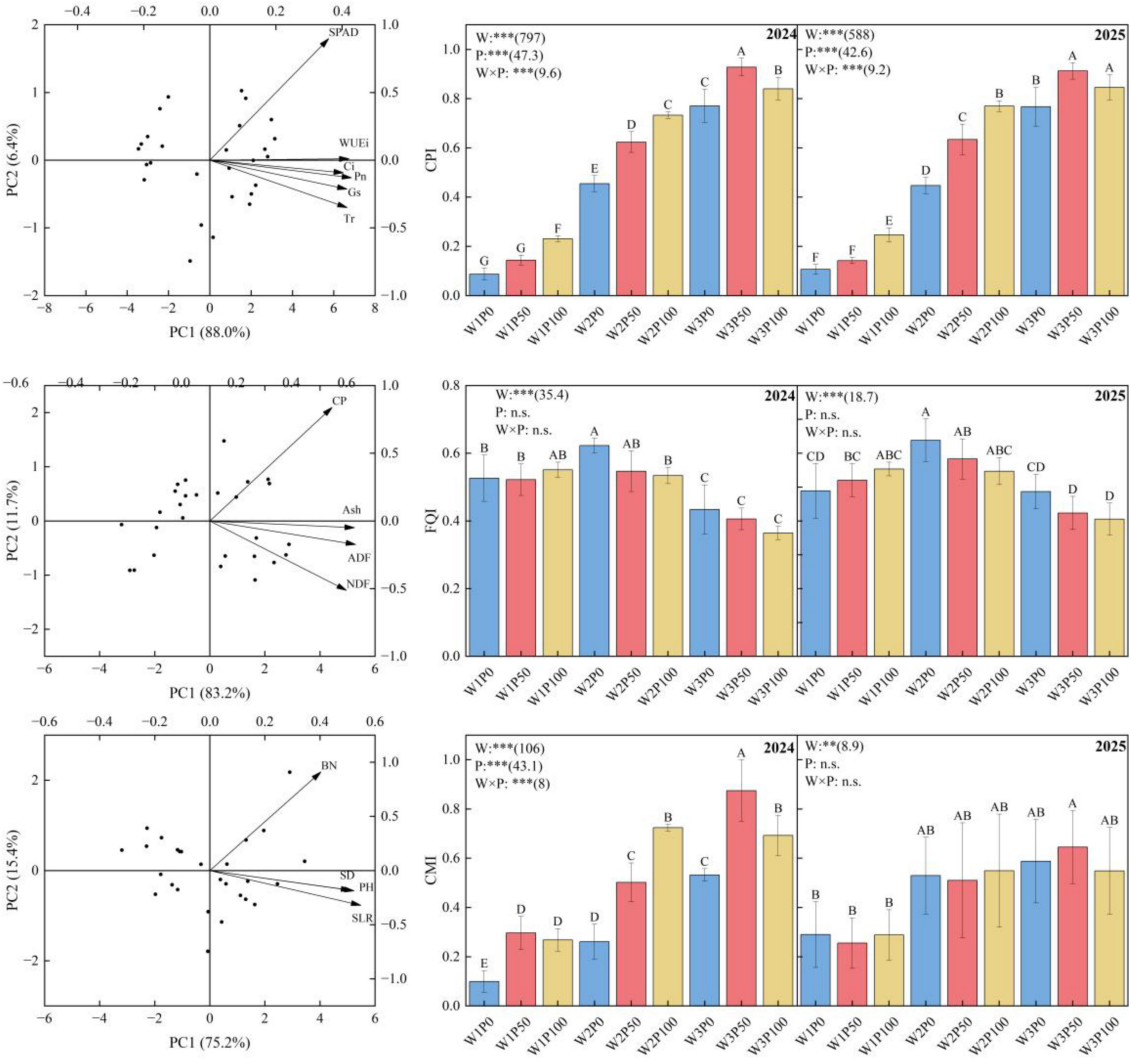
Principal component analysis of alfalfa photosynthetic characteristics, forage quality, and plant morphological traits, and effects of different irrigation and phosphorus fertilization combinations on comprehensive photosynthetic index (CPI), forage quality index (FQI), and comprehensive morphological index (CMI). Different uppercase letters indicate significant differences among treatments at (P< 0.05). W, and P represent the significance of irrigation and phosphorus fertilization respectively. *, **, ***, and n.s. indicate p< 0.05, p< 0.01, p< 0.001, and p > 0.05, respectively. Values in parentheses indicate F-values.

Principal component analysis was performed separately for photosynthetic characteristics, forage quality, and plant morphological traits (**Figure 8**). For photosynthetic characteristics, the first and second principal components explained 88.0% and 6.4% of the total variance, respectively, with a cumulative contribution of 94.4%. For forage quality, the first and second principal components explained 83.2% and 11.7% of the total variance, respectively, with a cumulative contribution of 94.9%. For plant morphological traits, the first and second principal components explained 75.2% and 15.4% of the total variance, respectively, with a cumulative contribution of 90.6%. W3P50 had the highest comprehensive photosynthetic index (CPI) and comprehensive morphological index (CMI). However, its forage quality index (FQI) was significantly lower than those under moderate and mild water stress treatments.

**Figure 9:**
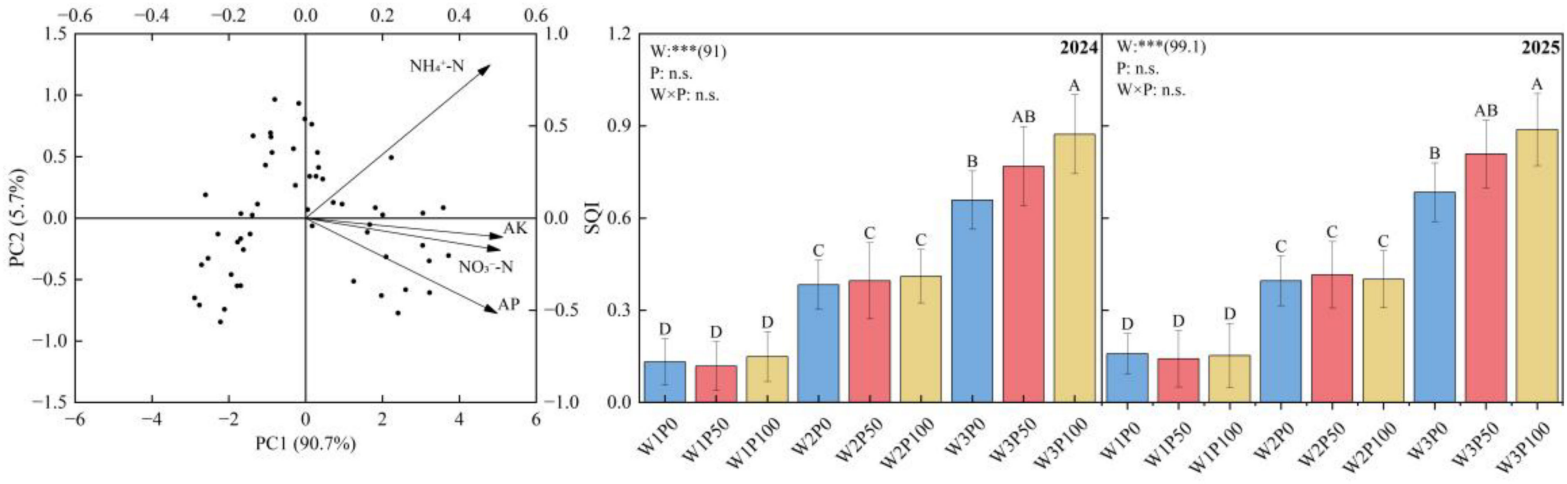
Effects of different irrigation and phosphorus fertilization combinations on soil quality index (SQI)under different irrigation and phosphorus fertilization combinations.

Soil quality index (SQI) differed significantly among irrigation treatments in both 2024 and 2025, while phosphorus fertilization and the W × P interaction were not significant (**Figure 9**). In 2024, SQI was highest under W3P100, followed by W3P50 and W3P0, whereas the W1 treatments showed the lowest values. The W2 treatments had intermediate SQI values and did not differ significantly among phosphorus levels. A similar pattern was observed in 2025, with the highest SQI recorded under W3P100 and W3P50, followed by W3P0, while all W1 treatments remained at the lowest level.

### 3.7 Integrated comparison of yield, resource-use efficiency, and comprehensive indices

The entropy-weighted TOPSIS results showed that the comprehensive performance of different irrigation and phosphorus fertilization treatments varied markedly (**Table 4**). W2P50 had the highest TOPSIS score (*C*_i_ = 0.6328) and ranked first, followed by W3P50 (C_i_ = 0.6076) and W2P100 (C_i_ = 0.5345). In contrast, the non-phosphorus treatments ranked relatively low, with W1P0 showing the lowest comprehensive score (C_i_ = 0.3012). Overall, treatments with moderate phosphorus fertilization, especially under mild water stress, showed better comprehensive performance than those without phosphorus application.

**Table 4:** Entropy-weighted TOPSIS scores and rankings of different irrigation and phosphorus fertilization treatments.

Treatment	TOPSIS score (*C*_i_)	Rank
W2P50	0.6328	1
W3P50	0.6076	2
W2P100	0.5345	3
W3P100	0.4691	4
W1P100	0.4581	5
W1P50	0.4366	6
W3P0	0.3405	7
W2P0	0.3089	8
W1P0	0.3012	9

## 4 Discussion

### 4.1 Effects of water–phosphorus coupling on yield and resource-use efficiency

This study demonstrated that irrigation level, phosphorus fertilization rate, and interannual variation jointly regulated alfalfa yield formation, irrigation water use efficiency (IWUE), and agronomic phosphorus efficiency (AEP) in an arid and semi-arid production system. Across two years and three cuttings, yield, IWUE, and AEP showed clear interannual and cutting-dependent variation, indicating that the response of alfalfa to water–phosphorus management was jointly controlled by annual climatic conditions and regrowth-stage growth environments (Miao et al., 2025). The significant irrigation × phosphorus interaction observed only in 2024 further suggests that the combined effect of water and phosphorus supply on yield and resource-use efficiency was more sensitive to annual environmental conditions in that year.

Overall, W2P50 and W3P50 consistently improved alfalfa yield compared with W1P50, particularly during the first cutting in 2024 and across the second and third cuttings in both years. After the first cutting in 2024, yield under W2P50 and W3P50 increased by 8.2% and 10.4%, respectively, compared with W1P50, while the corresponding increases in 2025 were 3.3% and 4.4%. These results indicate that mild water stress and full irrigation combined with moderate phosphorus fertilization promoted alfalfa regrowth and yield formation, although the magnitude of the yield response varied between years. This yield improvement can be attributed to the alleviation of water and phosphorus limitations, which helps maintain leaf gas exchange, photosynthetic carbon assimilation, canopy development, and post-cutting regrowth (Staniak et al., 2018). Previous studies have also shown that water deficit restricts alfalfa growth and forage yield, whereas increasing irrigation generally promotes biomass accumulation by improving plant water status and physiological activity (Carter and Sheaffer, 1983).

However, the higher yield under W2 and W3 was accompanied by a reduction in IWUE compared with W1, especially under P50 and P100 treatments. Annual yield under W2P50 and W3P50 was significantly higher than that under W1P50, and annual yield under W2P100 and W3P100 was significantly higher than that under W1P100. Nevertheless, IWUE under W2P50, W3P50, W2P100, and W3P100 was significantly lower than that under W1P50 and W1P100. This indicates that although additional irrigation increased hay yield, the yield gain was not sufficient to offset the increased irrigation input, resulting in lower IWUE under higher irrigation levels (Li and Su, 2017). Therefore, full irrigation may be beneficial when maximum forage yield is the primary objective, but it may not represent the most efficient strategy for water-limited production systems. Phosphorus fertilization also played an important role in regulating yield and phosphorus-use efficiency. In both years, AEP under W2P50 and W3P50 was significantly higher than that under W2P100 and W3P100, indicating that moderate phosphorus fertilization produced a greater yield return per unit phosphorus input than high phosphorus fertilization. In addition, AEP under W2P50 and W3P50 was significantly higher than that under W1P50 only in 2024, suggesting that the positive effect of improved irrigation on phosphorus agronomic efficiency was partly dependent on annual conditions. The absence of significant yield differences between W2P50 and W3P50 in most cases, together with the higher AEP under P50 than P100, suggests that moderate phosphorus fertilization can maintain relatively high alfalfa yield while improving phosphorus input efficiency.

Collectively, these results highlight a clear trade-off between yield improvement and resource-use efficiency under water–phosphorus coupling management. Although full irrigation increased yield, mild water stress combined with moderate phosphorus fertilization achieved a better balance between yield performance, IWUE, and AEP. Therefore, water–phosphorus management in alfalfa should shift from simply pursuing maximum yield to optimizing yield stability, water-use efficiency, and phosphorus input efficiency in an integrated manner (Djaman et al., 2020). Based on the present results, W2P50 can be considered a more suitable water-saving and phosphorus-efficient management option, whereas W3P50 may be used when the production objective is to maximize forage yield under relatively sufficient water availability.

### 4.2 Morphological and photosynthetic responses to irrigation and phosphorus fertilization

Alfalfa yield and plant growth traits showed strong cutting- and treatment-dependent responses (Varol et al., 2024). In the present study, irrigation significantly affected plant height, stem diameter, leaf-to-stem ratio, and branch number, indicating that water availability was a primary factor controlling canopy construction and aboveground structural development. In both 2024 and 2025, stem diameter under W2P50 and W3P50, as well as under W2P100 and W3P100, was significantly higher than that under W1P50 and W1P100, respectively. This suggests that mild water stress and full irrigation promoted stem development under phosphorus fertilization, thereby providing a stronger structural basis for biomass accumulation. In 2024, plant height under W2P50 and W3P50 was also significantly higher than that under W1P50, with no significant difference between W2P50 and W3P50. These responses may be attributed to the ability of adequate water supply to maintain cell expansion, assimilate transport, and post-cutting regrowth, whereas water limitation generally restricts stem elongation and canopy development (Meuriot et al., 2004; Zheng et al., 2023). The significant irrigation × phosphorus interaction for leaf-to-stem ratio only in 2024 further indicates that biomass allocation between leaves and stems was partly dependent on annual environmental conditions.

Increasing irrigation significantly improved alfalfa photosynthetic capacity, but its effects on resource-use efficiency were not always positive (Kuslu et al., 2010). Under the same phosphorus level, Pn, Tr, and Gs under W3 were significantly higher than those under W1 and W2 in both experimental years, indicating that full irrigation maintained greater stomatal opening, CO_2_ diffusion, transpiration, and photosynthetic carbon assimilation. These physiological responses likely contributed to the improvement in plant height, stem diameter, and yield under higher irrigation levels (Abid et al., 2016). The relatively strong photosynthetic and growth performance under W3P50 and W3P100 further suggests that higher water availability favored photosynthetic production capacity and aboveground morphological development (Hui et al., 2025). However, Pn did not differ significantly between W3P50 and W3P100, indicating that increasing phosphorus input from P50 to P100 did not further enhance photosynthetic capacity under full irrigation. This result supports the existence of a phosphorus response threshold under adequate water supply. Moreover, WUEi under W2P50 and W3P50 was significantly higher than that under W1P50, with no significant difference between W2P50 and W3P50. This indicates that moderate phosphorus fertilization improved leaf-level water-use performance under both mild water stress and full irrigation. Nevertheless, full irrigation may still reduce field-scale IWUE because the increase in yield may not fully offset the greater irrigation input (Li et al., 2023).

### 4.3 Phosphorus response threshold and forage quality regulation

Phosphorus fertilization significantly affected alfalfa yield, photosynthetic characteristics, and forage quality, but its effect depended strongly on irrigation level (Erkovan et al., 2022). Under moderate water stress, phosphorus fertilization partly alleviated the negative effects of water limitation on alfalfa growth and photosynthetic processes. This may be associated with the role of phosphorus in promoting root activity, energy metabolism, nutrient uptake, and photosynthate transport, thereby improving plant tolerance to stress conditions (Xu et al., 2025). However, under mild water stress and full irrigation, yield, IWUE, and several photosynthetic traits showed limited differences between P50 and P100. Across irrigation and phosphorus treatments, yield was highest under W3P50, with no significant difference from W3P100. Under P50, W2P50 and W3P50 increased yield by 5.3% and 7.9%, respectively, compared with W1P50, whereas under P100, W2P100 and W3P100 increased yield by 4.0% and 5.0%, respectively, compared with W1P100. These results indicate that the yield response to phosphorus fertilization was not linear, but showed a threshold regulated by water availability. Excessive phosphorus input may fail to further increase yield and may instead reduce phosphorus agronomic efficiency. Therefore, under relatively suitable water conditions, reduced phosphorus fertilization is feasible for maintaining yield while improving phosphorus-use efficiency (Zhang et al., 2020b).

The response of forage quality to water–phosphorus coupling was not fully consistent with that of yield and morphological growth (Hu et al., 2021). Irrigation and phosphorus fertilization significantly affected crude protein, ash, NDF, and ADF, indicating that both water and phosphorus supply were important regulators of alfalfa nutritive composition. In both years, CP under W2P50 and W3P50 was significantly higher than that under W1P50, increasing by 11.9% and 11.5% in 2024 and by 12.1% and 11.9% in 2025, respectively. These results suggest that moderate phosphorus fertilization combined with improved water supply enhanced nitrogen assimilation and protein accumulation in alfalfa tissues. Mild water stress may maintain relatively high crude protein content by limiting excessive vegetative growth and reducing the dilution effect of structural biomass accumulation (Liu et al., 2018). In contrast, full irrigation can promote plant height, stem diameter, and yield, but may also increase structural carbohydrate accumulation, thereby affecting forage nutritive value (Ball et al., 2001). In the present study, compared with W1P50, only W2P50 significantly increased ADF and ash, whereas NDF did not differ significantly. This indicates that the improvement in CP under W2P50 was accompanied by some changes in fiber and mineral composition, rather than a uniform improvement in all forage quality traits. Therefore, forage quality under water–phosphorus coupling should be evaluated comprehensively instead of relying on a single indicator (Linn and Martin, 1991).

### 4.4 Soil nutrient availability and potential nitrate leaching risk

However, the potential risk of nitrate leaching under different irrigation regimes should also be considered. Nitrate is highly mobile in soil and can move downward with irrigation water, especially when water input exceeds crop uptake and soil water-holding capacity (Cameron et al., 2013). In the present study, full irrigation increased soil nutrient availability and supported higher photosynthetic capacity and yield, but the greater water input under W3 may also increase the possibility of downward NO_3_^-^-N transport, particularly in the deeper 40–60 cm soil layer. Although alfalfa has a deep root system and can utilize nitrate from deeper soil layers, excessive irrigation may still promote nitrate movement beyond the main root zone if residual soil nitrate is not fully absorbed (Singh et al., 2023). Therefore, while W3P50 can be used as a yield-prioritized option, its potential nitrate leaching risk should be considered in water-limited and environmentally sensitive regions. This further supports the recommendation of W2P50 as a more balanced strategy for maintaining yield while reducing water input and potential nutrient loss. Future studies should directly monitor soil water movement, nitrate distribution below 60 cm, and leachate nitrate concentration to better evaluate the environmental effects of water–phosphorus coupling management.

### 4.5 Integrated evaluation and optimized water–phosphorus management strategy

Comprehensive evaluation further revealed functional differentiation among water–phosphorus combinations. W3P50 produced the highest yield and showed strong photosynthetic and growth performance, but W2P50 obtained the highest entropy-weighted TOPSIS score and ranked first among all treatments. This indicates that the treatment with the highest yield was not necessarily the treatment with the best overall performance. The entropy-weighted TOPSIS results showed that W2P50 had the highest comprehensive score, followed by W3P50 and W2P100, whereas non-phosphorus treatments ranked relatively low, especially W1P0. This suggests that moderate phosphorus fertilization, especially under mild water stress, achieved a better balance among productivity, forage quality, physiological performance, water-use efficiency, phosphorus-use efficiency, and soil nutrient status. These results are consistent with the idea that different comprehensive indices represent different production objectives: full irrigation combined with moderate phosphorus fertilization corresponds to a “high photosynthesis–strong growth–high yield” pathway, whereas mild water stress combined with moderate phosphorus fertilization represents a “water saving–phosphorus reduction–high quality–high efficiency” pathway (Cao et al., 2025; Gallo et al., 2013).

Soil available nutrient status also contributed to the overall response of alfalfa to water–phosphorus coupling (Wang et al., 2021). SQI under phosphorus fertilization in the W3 treatment was significantly higher than that under W2 and W1, and the irrigation × phosphorus interaction significantly affected SQI. This indicates that soil nutrient availability was jointly regulated by water and phosphorus supply. Irrigation can influence nutrient dissolution, diffusion, mass flow, and vertical redistribution in the soil profile, whereas phosphorus fertilization directly improves soil available phosphorus supply. The higher SQI under W3 with phosphorus fertilization suggests that sufficient water supply enhanced the effectiveness of phosphorus input in improving soil nutrient availability. Improved soil nutrient status may further support root nutrient uptake, leaf photosynthesis, canopy development, and yield formation. In particular, topsoil nutrient availability often plays an important role in supporting rapid alfalfa growth and physiological activity (Kong et al., 2020). Meanwhile, nutrient supply in deeper soil layers may also contribute to sustained growth and post-cutting regrowth because alfalfa has a deep root system and can utilize water and nutrients from the subsoil (Berg et al., 2005). Overall, water–phosphorus coupling enhanced soil available nutrient supply, which further improved leaf photosynthetic capacity and plant morphological development, ultimately driving alfalfa yield and quality formation (Liu et al., 2021b).

Based on the multi-year results and comprehensive evaluation, mild water stress combined with moderate phosphorus fertilization represents a more integrated water–phosphorus management strategy for alfalfa production in arid and semi-arid regions (Hanson et al., 2007). Although W3P50 produced the highest yield, its advantage over W2P50 was limited, whereas W2P50 showed higher comprehensive performance in terms of AEP, IWUE, forage quality, and overall TOPSIS ranking. This suggests that W2P50 can maintain stable yield while improving water- and phosphorus-use efficiency under reduced resource inputs. In contrast, W3P50 is more suitable when the main production objective is to maximize hay yield and irrigation water is relatively sufficient. Therefore, management decisions in water- and phosphorus-limited regions should not focus solely on maximum yield, but should place greater emphasis on yield and quality returns per unit resource input. Future studies should further incorporate root distribution, soil water dynamics, phosphorus migration, nutrient balance after long-term repeated cutting, and economic evaluation to determine the stability and environmental effects of different water–phosphorus management strategies in long-term alfalfa production systems (Zhang et al., 2025).

## 5 Conclusions

This study demonstrated that water–phosphorus coupling regulated the trade-off among yield, forage quality, and resource-use efficiency in alfalfa production under arid and semi-arid conditions. Full irrigation combined with moderate phosphorus fertilization enhanced photosynthetic capacity, morphological growth, and hay yield, whereas mild water stress combined with moderate phosphorus fertilization improved irrigation water use efficiency, phosphorus agronomic efficiency, and forage quality while maintaining relatively stable yield. The entropy-weight TOPSIS evaluation further confirmed that W2P50 achieved the best overall performance across yield, forage quality, and resource-use efficiency indicators. Therefore, W2P50 can be recommended as an optimized water–phosphorus management strategy for water-saving, phosphorus-efficient, high-quality, and stable-yield alfalfa production in arid and semi-arid regions. When the primary objective is maximum hay yield and water resources are sufficient. Future studies should further evaluate the long-term stability and environmental effects of this strategy under repeated cutting and multi-year production conditions.

## Data Availability

The raw data supporting the conclusions of this article will be made available by the authors, without undue reservation.
