# Supplementary material for: Irrigation and phosphorus fertilization regulate trade-offs among yield, forage quality, and resource-use efficiency in alfalfa production under arid and semi-arid conditions
Source: Front Plant Sci. 2026 Jul 29;17:1885390. doi: 10.3389/fpls.2026.1885390 (PMC13461903; doi:10.3389/fpls.2026.1885390)
Supplement: Supplementary file 1 [file Table1.docx]

**Table S1.** Irrigation frequency and total irrigation amount under different irrigation treatments across three cuttings of alfalfa.

| **Cutting** | **Irrigation treatment** | **Irrigation frequency** | **Total irrigation amount (m³ ha⁻¹)** |
| --- | --- | --- | --- |
| First cutting | W1 | 3 | 501 |
| First cutting | W2 | 3 | 604.5 |
| First cutting | W3 | 3 | 661.5 |
| Second cutting | W1 | 3 | 493.5 |
| Second cutting | W2 | 3 | 591 |
| Second cutting | W3 | 3 | 672 |
| Third cutting | W1 | 2 | 322.5 |
| Third cutting | W2 | 2 | 385.5 |
| Third cutting | W3 | 2 | 436.5 |

**Table S2** PCA-derived weighting coefficients for the comprehensive photosynthetic index, forage quality index, and comprehensive morphological index.

| **Composite index** | **Indicator** | **PCA1 coefficient** | **Normalized weight** |
| --- | --- | --- | --- |
| CPI | Pn | 0.42905 | 0.17543 |
| CPI | Tr | 0.41564 | 0.16995 |
| CPI | Gs | 0.41518 | 0.16976 |
| CPI | Ci | 0.40457 | 0.16542 |
| CPI | WUEi | 0.42149 | 0.17234 |
| CPI | SPAD | 0.35979 | 0.14711 |
| FQI | CP | 0.44423 | 0.22263 |
| FQI | Ash | 0.52535 | 0.26329 |
| FQI | NDF | 0.49618 | 0.24867 |
| FQI | ADF | 0.52959 | 0.26541 |
| CMI | PH | 0.52387 | 0.26356 |
| CMI | SD | 0.51176 | 0.25746 |
| CMI | SLR | 0.54836 | 0.27588 |
| CMI | BN | 0.4037 | 0.2031 |
